# Isovaleric acid ameliorates ovariectomy‐induced osteoporosis by inhibiting osteoclast differentiation

**DOI:** 10.1111/jcmm.16482

**Published:** 2021-03-25

**Authors:** Kwang Min Cho, Ye Seon Kim, Mingyu Lee, Ha Young Lee, Yoe‐Sik Bae

**Affiliations:** ^1^ Department of Biological Sciences Sungkyunkwan University Suwon Korea; ^2^ Department of Health Sciences and Technology SAIHST Sungkyunkwan University Seoul Korea

**Keywords:** branched‐chain fatty acid, isovaleric acid, macrophage, osteoclast, osteoporosis

## Abstract

Osteoclasts (OCs) play important roles in bone remodelling and contribute to bone loss by increasing bone resorption activity. Excessively activated OCs cause diverse bone disorders including osteoporosis. Isovaleric acid (IVA), also known as 3‐methylbutanoic acid is a 5‐carbon branched‐chain fatty acid (BCFA), which can be generated by bacterial fermentation of a leucine‐rich diet. Here, we find that IVA suppresses differentiation of bone marrow‐derived macrophages into OCs by RANKL. IVA inhibited the expression of OC‐related genes. IVA‐induced inhibitory effects on OC generation were attenuated by pertussis toxin but not by H89, suggesting a G_i_‐coupled receptor‐dependent but protein kinase A‐independent response. Moreover, IVA stimulates AMPK phosphorylation, and treatment with an AMPK inhibitor blocks IVA‐induced inhibition of OC generation. In an ovariectomized mouse model, addition of IVA to the drinking water resulted in significant decrease of body weight gain and inhibited the expression of not only OC‐related genes but also fusogenic genes in the bone tissue. IVA exposure also blocked bone destruction and OC generation in the bone tissue of ovariectomized mice. Collectively, the results demonstrate that IVA is a novel bioactive BCFA that inhibits OC differentiation, suggesting that IVA can be considered a useful material to control osteoclast‐associated bone disorders, including osteoporosis.

## 1 INTRODUCTION

Multinucleated osteoclasts (OCs) can be differentiated from the macrophage lineage.^1^, ^2^ OCs play important roles to regulate bone metabolism and homeostasis with OC‐specific enzymes such as cathepsin K (CTSK) and tartrate‐resistant acid phosphatase (TRAP).^2^ OCs can absorb the osteoid and dissolve the hydroxyapatite crystals, leading to diminished bone mineral density and bone growth.^2^, ^3^ Several pathological conditions including postmenopausal osteoporosis are caused by reinforced OC activity.^4^ Therefore, modulation of OC differentiation and activity is crucial to maintain bone homeostasis.

Five or fewer carbons containing fatty acid are called short‐chain fatty acids (SCFAs). The SCFAs are mainly derived from gut microbiota and regulate diverse pathophysiological responses in several different organs and tissues after being absorbed by the intestine.^5^, ^6^ For example, previous reports demonstrated that several SCFAs regulate immune cell functions by regulating cytokine and chemokine production.^7^ SCFAs modulate neutrophil recruitment by regulating the expression of cytokine‐induced neutrophil chemoattractant‐2αβ and the adhesion molecule L‐selectin.^8^ SCFAs also regulate cellular differentiation.^9^, ^10^ Especially, butyric acid induces apoptosis to inhibit macrophage differentiation.^11^ Dendritic cells treated with butyric acid promote regulatory T‐cell differentiation from naïve T cells.^12^ Regarding the target receptors for the SCFAs, several G‐protein coupled receptors (GPCRs) including Olfr78 have been reported to mediate biological responses caused by the SCFAs.^7^, ^13^, ^14^

Branched‐chain fatty acids (BCFAs) are made from essential amino acids including leucine by microbial fermentation, to form isobutyric acid with four carbons, or 2‐methylbutyric acid and isovaleric acid (IVA) with five carbons.^15^, ^16^ The levels of the leucine metabolite IVA are increased by the consumption of high protein foods and certain pathological conditions including colon cancer.^16^, ^17^ Previous reports showed that BCFAs affect energy metabolism and cause colon smooth muscle relaxation.^18^, ^19^ Another report demonstrated that enterochromaffin cells are activated by a microbial metabolite, IVA, via Olfr558.^20^ However, much remains unknown regarding whether BCFAs regulate additional cellular activities and differentiation.

In this study, we examined whether IVA affects the activity of macrophages and their differentiation into OCs. The molecular mechanism involved in the modulation of OC differentiation by IVA was investigated as well. We also examined the in vivo effects of IVA on the regulation of osteoporosis, an OC‐associated disorder.

## 2 MATERIALS AND METHODS

### 2.1 Generation of mouse bone marrow‐derived macrophages (BMDMs)

The protocols for mouse experiments received the approval of the Institutional Review Committee for Animal Care and Use at Sungkyunkwan University (Suwon, Korea). Mouse BMDMs were generated as described before.^21^ Briefly, total bone marrow cells were obtained from the femur and tibia of 5‐week‐old C57BL/6 mice (Orient Bio). After culturing the bone marrow cells in α‐MEM (Gibco) with 10% FBS (Access Biological) for 1 day, non‐adherent cells were further cultured for 3 days in α‐MEM with 10% FBS and 30 ng/mL of M‐CSF (Peprotech).

### 2.2 OC differentiation and TRAP staining

Isolated mouse BMDMs (1 × 10^4^ cells/well) were cultured with 30 ng/mL of M‐CSF and 100 ng/mL of RANKL (Peprotech) in 96‐well plates for 3 days.^22^ Differentiated immature OCs were further differentiated into mature OCs by adding α‐MEM with 10% FBS, 30 ng/mL of M‐CSF and 100 ng/mL of RANKL for 2 days. Before TRAP staining, mature OCs were fixed with paraformaldehyde (4%) for 20 minutes, as described previously.^23^ Stained multinuclear cells (≥3nuclei) were considered to be OCs and were counted.

### 2.3 Cell viability assay

To determine cell viability, BMDMs were treated by IVA (Sigma‐Aldrich), and culture media were applied to the LDH assay kit (Promega) at 490 nm.

### 2.4 Chemotaxis assay

Chemotactic migration of BMDMs was conducted using multi‐well chambers (Neuroprobe Inc) as reported previously.^24^ Briefly, 30 μL of chemoattractants such as IVA and WKYMVm (Anygen) were loaded on the lower well and then 25 μL of suspended BMDMs (1 × 10^6^ cells/mL) in α‐MEM were applied on the upper well, separated by a polyhydrocarbon filter. After 2 hours, cells that had migrated through the filter were stained with haematoxylin. Migrated cells in each well were counted under a microscope (Leica DM750).

### 2.5 Western blot analysis

BMDMs were stimulated with 30 ng/mL of M‐CSF and 100 ng/mL of RANKL in the absence or presence of 200 μM of IVA for the indicated lengths of time. Extracted proteins were separated by 10% SDS‐PAGE and transferred to a 0.45 μm nitrocellulose membrane (Cytiva). After incubating the membranes with antibodies for target proteins, visualization with enhanced chemiluminescence followed. Antibodies used for Western blot analyses were obtained from Cell Signalling Technology or Santa Cruz Biotechnology.

### 2.6 Bone resorption assay

The pit formation assay was performed on Corning 96‐well plates (Corning). BMDMs (1 × 10^4^ cells/well) were cultured with 30 ng/mL of M‐CSF and 100 ng/mL of RANKL in 96‐well plates for 7 days. The media containing M‐CSF and RANKL was changed every 2 or 3 days after seeding the cells. After 7 days of induction, the media was removed and cells were incubated in 100 μL of 10% bleach solution for 5 minutes at room temperature. Wells were washed with dH_2_O 2 times and dried at room temperature for 4 hours. The pit clusters generated by OCs were photographed by a microscope.

### 2.7 RNA isolation

Total RNA from mouse BMDMs and immature OCs were isolated using TRIzol reagent (Invitrogen). cDNAs were synthesized using the RT Premix Kit (iNtRON). Femurs and tibias of ovariectomy (OVX) mouse models were rapidly frozen in liquid nitrogen and ground in a homogenizer to extract RNA using TRIzol reagent.

### 2.8 Quantitative polymerase chain reaction (qPCR) analysis

qPCR analyses were conducted using the Rotor‐gene Q (2plex on PC) instrument (QIAGEN) with SYBR Green qPCR Mix (Biofact). The primers used for qPCR analyses were presented in Table 1. Data were normalized against the expression of *GAPDH* as an internal control.

**TABLE 1:** Primers used for qPCR analysis

*RANK*	5′‐AGAAGACGGTGCTGGAGTCT‐3′	Forward
	5′‐TAGGAGCAGTGAACCAGTCG‐3′	Reverse
*TRAF6*	5′‐GCCCAGGCTGTTCATAATGT‐3′	Forward
	5′‐TCGCCCACGTA CATACTCTG‐3′	Reverse
*OSCAR*	5′‐CTGCTGGATACGGATCAGCTCCCCAGA‐3′	Forward
	5′‐CCAAGGAGCCAGAACCTTCGAAACT‐3′	Reverse
*TRAP*	5′‐CAGTTGGCAGCAGCCAAGGAGGAC‐3′	Forward
	5′‐TCCGRGCTCGGCGATGGACCAGA‐3′	Reverse
*Blimp1*	5′‐TGCTTATCCCA GCACCCC‐3′	Forward
	5′‐ CTTCAGGTTGGAGAGCTGACC −3′	Reverse
*c‐fos*	5′‐AGAGCGGGAATGGTGAAGAC‐3′	Forward
	5′‐GCTGCATAGAAGGAACCGGA‐3′	Reverse
*NFATc1*	5′‐ CAACGCCCTGA CCACCGATAG −3′	Forward
	5′‐GGGAAGTCAGAAGTGGGTGGA‐3′	Reverse
*Ctsk*	5′‐GGGAGAAAAACCTGAAGC‐3’	Forward
	5′‐ATTCTGGGGACTCAGAGC‐3’	Reverse
*MafB*	5′‐AGTGTGGAGGACCGCTTCTCT‐3′	Forward
	5′‐CAGAAAGAACTCAGGAGAGGAGG‐3′	Reverse
*IRF8*	5′‐AGACGAGGTTACGCTGTGC‐3′	Forward
	5′‐ TCGGGGACAATTCGGTAAACT −3′	Reverse
*OC‐STAMP*	5′‐TGGGCCTCCATATGACCTCGAGTAG‐3′	Forward
	5′‐TCAAAGGCTTGTAAATTGGAGGAGT‐3′	Reverse
*DC‐STAMP*	5′‐GGGTGCTGTTTGCCGCTG‐3′	Forward
	5′‐CGACTCCTTGGGTTCCTTGCT‐3′	Reverse
*Atp6v0d2*	5′‐TTCTTGAGTTTGAGGCCGAC‐3′	Forward
	5′‐CAGCTTGAGCTAACAACCGC‐3′	Reverse
*PGC‐1α*	5′‐ AGCACACGTTTATTCACGGGT‐3′	Forward
	5′‐ GCCCCCAAGTCCTCACATG‐3′	Reverse
*ALP*	5′‐TGAATGACGGGCCTGATGAC‐3′	Forward
	5′‐GGTACTTATCCCGGGCCTTG‐3′	Reverse
*Osx*	5′‐AAAGGAGGCACAAAGAAGC‐3′	Forward
	5′‐CAGGAAATGAGTGAGGGAAG‐3′	Reverse
*Runx2*	5′‐CCCTGAACTCTGCACCAAGT‐3′	Forward
	5′‐TGGAGTGGATGGATGGGGAT‐3′	Reverse
*GAPDH*	5′‐ CCACCACCCTGTTGCTGTA‐3′	Forward
	5′‐ AATGTGTCCGTCGTGGATCT‐3′	Reverse

### 2.9 OVX mouse model

OVX was performed with 8‐week‐old C57BL/6 female mice according to a previous report.^25^ Mice were anaesthetized by inhalation anaesthesia, and ovaries were bilaterally removed via a dorsal approach as described previously.^26^ To examine the effects of IVA on OVX mice, we added IVA to the drinking water (at a final concentration of 75 mM or 150 mM) from the day after surgery. The mice were sacrificed after 5 weeks, and the femurs were separated and used in subsequent experiments.

### 2.10 Bone histology in OVX mice

All OVX model mice were sacrificed 5 weeks after surgery, and their bones were fixed in paraformaldehyde solution (4%) for 2 days. Next, the bones were decalcified in 10% EDTA solution for 3 weeks and embedded with paraffin. Bones were sectioned into 4 μm by a microtome. After staining the samples with haematoxylin and eosin (H&E) solution, morphological analysis such as bone density was conducted. TRAP staining was conducted to determine OC formation.

### 2.11 Statistical analysis

GraphPad Prism software was used to evaluate results. Statistical analysis was performed using Student's *t*‐test or ANOVA (one‐way or two‐way). All results are expressed as the mean ± SEM. A *P*‐value < .05 was considered statistically significant.

## 3 RESULTS

### 3.1 IVA stimulates BMDM migration in a pertussis toxin (PTX)‐sensitive manner

**FIGURE 1:**
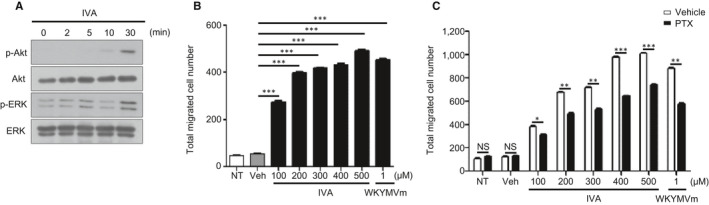
IVA stimulates BMDMs leading to chemotactic migration. A, BMDMs were stimulated with IVA (200 μM) for several lengths of time. Levels of phosphorylated‐Akt, phosphorylated‐ERK, Akt and ERK were determined by Western blot. B, BMDMs were applied to multi‐well Boyden chambers containing different concentrations of IVA (0, 100, 200, 300, 400, and 500 μM) or WKYMVm (1 μM) for 2 h. Total migrated cell numbers were counted under a microscope. (C) After incubating BMDMs with/without PTX (10 ng/mL) for 4 h, the incubated cells were applied to a chemotaxis assay with IVA (0, 100, 200, 300, 400, and 500 μM) or WKYMVm (1 μM) for 2 h. Data are presented as the mean ± SEM (n = 3 for B, C). ^*^ *P* < .05, ^**^ *P* < .01, ^***^ *P* <.001. NS: not significant

Various extracellular stimuli elicit the activation of crucial kinases including Akt and MAPKs during cellular activation.^27^, ^28^ In this study, we investigated whether IVA stimulates BMDMs by measuring the phosphorylation of Akt and ERK. Stimulation of BMDMs with IVA caused apparent phosphorylation of Akt and ERK at 30 minutes (Figure 1A). These results suggested that IVA may activate the BMDMs. Chemotactic migration is one of the important functional activities of BMDM, which is needed for subsequent cellular responses.^29^, ^30^ Next, we investigated the effects of IVA on macrophage chemotaxis at several different concentrations. Addition of IVA significantly elicited BMDM chemotactic migration, in an IVA concentration‐dependent manner (Figure 1B), maximal migration‐inducing activity was observed at 500 μM IVA, which is comparable to that of a well‐known macrophage chemoattractant, WKYMVm (Figure 1B). Previously, macrophage chemotactic migration was reported to be regulated by PTX‐sensitive GPCRs.^31^ We also examined the possible role of PTX‐sensitive GPCRs in IVA‐induced BMDM chemotactic migration. Addition of PTX to BMDMs prior to the chemotaxis assay with IVA resulted in significant inhibition of BMDM chemotactic migration (Figure 1C). These results suggest that IVA stimulates BMDMs leading to chemotactic migration in a PTX‐sensitive manner.

### 3.2 IVA inhibits RANKL‐induced OC differentiation

**FIGURE 2:**
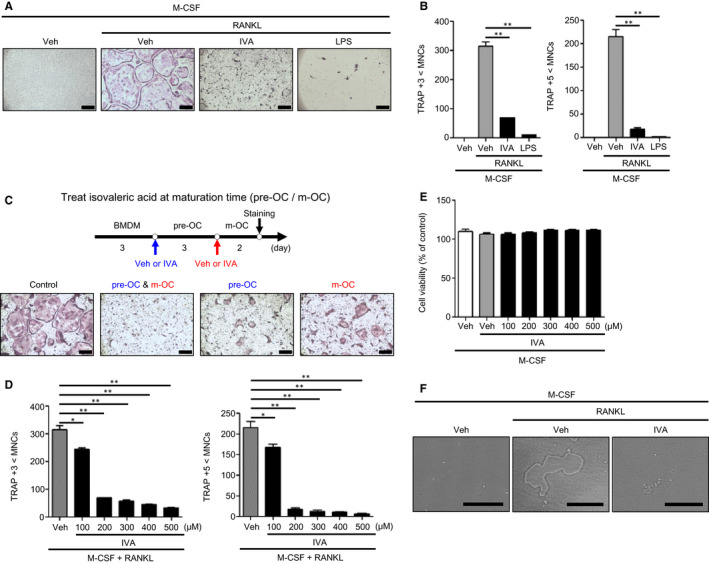
IVA blocks OC differentiation induced by RANKL. A, B, BMDMs were stimulated with IVA (200 μM) or LPS (1 μg/mL) under OC differentiation conditions (30 ng/mL M‐CSF plus 100 ng/mL RANKL) for 5 days. TRAP^+^ MNCs (>3 nuclei or >5 nuclei) were counted and considered to be OCs. C, A protocol to test the effects of IVA on OC generation at different time points (top). IVA was added at day 3 of BMDMs and/or at day 6 of pre‐OCs during osteoclastogenesis. Mature OCs were stained by TRAP staining solution (bottom). D, BMDMs were stimulated with IVA (0, 100, 200, 300, 400, and 500 μM) under OC differentiation conditions (30 ng/mL M‐CSF plus 100 ng/mL RANKL) for 5 days. With TRAP staining, the TRAP^+^ MNCs (>3 nuclei or >5 nuclei) were counted and considered to be OCs. (E) BMDMs were stimulated with IVA (0, 100, 200, 300, 400, and 500 μM) in the presence of M‐CSF (30 ng/mL) for 3 days. After harvesting the supernatant, the levels of LDH released were monitored by measuring ODs at 490 nm. (F) Representative images of bone resorption area on the Corning Osteo Assay Plate. BMDMs were stimulated with M‐CSF (30 ng/mL) and RANKL (100 ng/mL) with or without IVA (200 μM) for 7 days. Data are representative of three independent experiments (A, C, F). Data are presented as the mean ± SEM (n = 3 for B, D, E). ^*^ *P* < .05, ^**^ *P* < .01. Scale bar, 2 μm (A, C bottom). Scale bar, 100 μm (F)

Since IVA stimulates the activation of macrophages, which can differentiate into OCs, the effects of IVA on OC generation induced by RANKL was examined. Addition of M‐CSF and RANKL to BMDMs generated multinucleated OCs, which can be observed by TRAP staining (Figure 2A). Under OC differentiation conditions, 200 μM IVA showed a strong inhibitory effect on osteoclastogenesis (Figure 2A). Multinucleated TRAP‐positive OC numbers were significantly decreased upon IVA exposure (Figure 2B). LPS, a well‐known inhibitor of osteoclastogenesis, also significantly blocked OC differentiation (Figure 2A,B).

We compared the effects of IVA on OC generation at different time points in osteoclastogenesis (Figure 2C top). Addition of IVA at the pre‐OC stage significantly inhibited RANKL‐induced OC generation (Figure 2C bottom). The inhibitory effects of IVA on OC generation were stronger when IVA was added at the BMDM stage. Moreover, subsequent addition of IVA at the BMDM stage and the pre‐OC stage caused an almost complete inhibition of OC differentiation induced by RANKL (Figure 2C bottom). The results suggest that IVA can effectively inhibit osteoclastogenesis by working on at least two different stages of OC differentiation, and stronger inhibitory effects can be elicited at earlier stages of osteoclastogenesis. Concentration‐dependency analysis with two subsequent additions of IVA showed that IVA elicits slight inhibitory activity at 100 μM, and almost complete inhibitory activity at over 200 μM (Figure 2D). In order to check whether IVA inhibits OC generation by inducing cell death, we conducted a cell viability analysis and found that IVA did not affect cell viability during OC generation at 100 ~ 500 μM (Figure 2E). Collectively, IVA strongly inhibits RANKL‐induced OC differentiation without affecting cell viability. Mature OCs show bone resorbing activity.^32^, ^33^ Since we found that IVA suppressed OC formation, we next tested the effects of IVA on OC function by using dentine slices. We found that OC generated by M‐CSF + RANKL showed strong bone resorbing activity, while addition of IVA in the presence of M‐CSF + RANKL blocked this activity (Figure 2F). Taking our results together, IVA appears to block OC differentiation, leading to inhibition of bone resorbing activity.

### 3.3 IVA inhibits OC‐related gene expression

**FIGURE 3:**
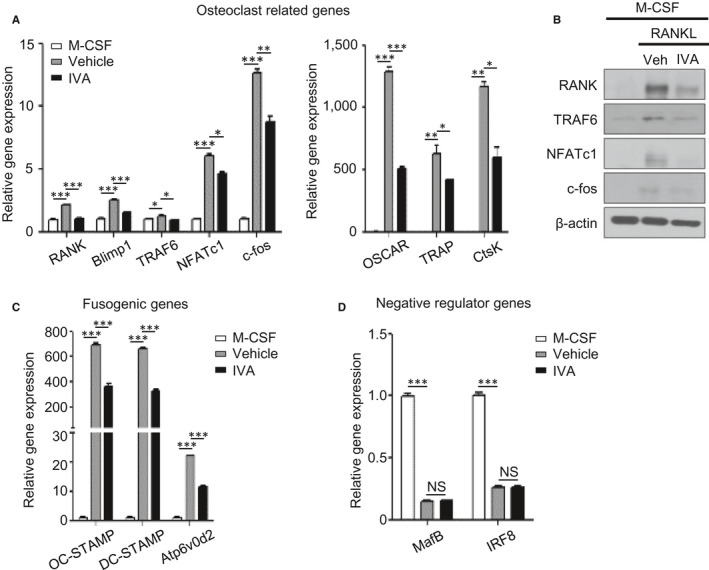
IVA regulates the expression of OC‐related genes induced by RANKL. A, C, D, Isolated BMDMs were treated with IVA (200 μM) in the presence of M‐CSF (30 ng/mL) and RANKL (100 ng/mL) for 3 days. After RNA isolation, qPCR analyses were conducted using specific primers for OC‐related genes (*RANK*, *Blimp1*, *TRAF6*, *NFATc1*, *c‐fos*, *OSCAR*, *TRAP* and *CtsK*) (A), fusogenic genes (*OC‐STAMP*, *DC‐STAMP* and *Atp6v0d2*) (C), and negative regulator genes of osteoclastogenesis (*MafB* and *IRF‐8*) (D) and *GAPDH*. (B) Western blot analysis was performed using antibodies against RANK, TRAF6, NFATc1, c‐fos, and β‐actin. Data are representative of three independent experiments (B). Data are presented as the mean ± SEM (n = 3 for A, C, D). ^*^ *P* < .05, ^**^ *P* < .01, ^***^ *P* < .001. NS: not significant

Our finding that IVA suppresses the differentiation of BMDMs into OCs led us to investigate if IVA affects the expression of OC‐related genes. RANKL significantly augmented mRNA expression of OC‐related genes including *RANK*, *Blimp1*, *TRAF6*, *c‐fos* and *NFATc1*, a master transcriptional factor of the OCs^34^ (Figure 3A left). Addition of IVA to differentiation conditions significantly decreased the expression of OC‐related genes (Figure 3A left). An additional set of OC‐related genes including *OSCAR*, *TRAP* and *Ctsk* was also significantly suppressed by IVA (Figure 3A right). IVA strongly inhibits the protein expression of OC‐related genes such as RANK, TRAF6, NFATc1 and c‐fos (Figure 3B). IVA also downregulated the expression of fusogenic genes including *OC‐STAMP*, *DC‐STAMP* and *Atp6v0d2* ^35^, ^36^ (Figure 3C). However, IVA did not affect the expression of negative regulator genes of osteoclastogenesis such as *MafB* and *IRF8* ^37^, ^38^ (Figure 3D). Together, these results indicate that IVA inhibits OC generation by suppressing the expression of not only OC‐related genes but also fusogenic genes.

### 3.4 IVA‐induced inhibitory effects on OC generation are partly mediated by PTX‐sensitive signalling and AMPK activity

**FIGURE 4:**
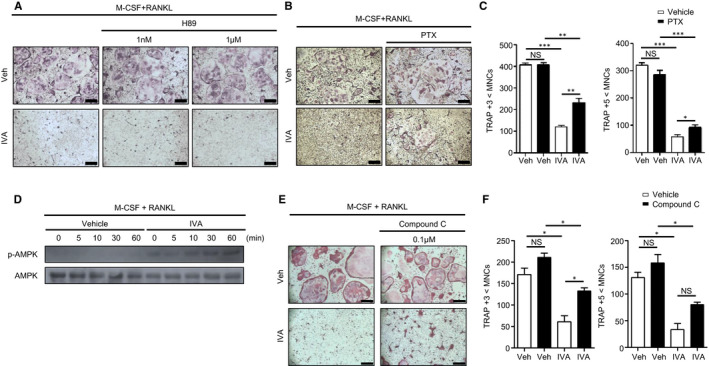
IVA inhibits RANKL‐induced osteoclastogenesis via G_i_‐coupled GPCR and AMPK. A‐C, E, F, Mouse BMDMs were stimulated with IVA (200 μM) in the absence or presence of H89 (1 nM or 1 μM) (A), the G_i_ inhibitor PTX (10 ng/mL) (B, C), compound C (0.1 μM) (E) under OC differentiation conditions (30 ng/mL M‐CSF plus 100 ng/mL RANKL) for 5 days. With TRAP staining, TRAP^+^ MNCs (>3 nuclei or >5 nuclei) were counted and considered to be OCs (C, F). D, BMDMs stimulated with IVA (200 μM) during each time point in OC differentiation condition media. Extracted proteins were separated by 10% SDS‐PAGE, and levels of phosphorylated‐AMPK and AMPK were determined by Western blot. Data are representative of at least three independent experiments (A, B, D, E). Data are presented as the mean ± SEM (n = 3 for C, F). ^*^ *P* < .05, ^**^ *P* < .01, ^***^ *P* < .001. NS: not significant. Scale bar, 2 μm (A, B, E)

Since IVA has been reported to be recognized by Olfr558,^20^ and odorant receptors signal through the cAMP/protein kinase A (PKA) pathway,^19^ we examined whether IVA inhibits OC generation through PKA. Preincubation of BMDMs with a PKA inhibitor (H89) prior to IVA treatment did not block IVA‐induced suppression of OC generation (Figure 4A). These results suggest that IVA inhibits OC generation in a cAMP/PKA‐independent manner. Next, we checked whether PTX‐sensitive signalling is involved in the IVA‐induced inhibitory effects on OC generation, because IVA‐induced BMDM chemotactic migration is blocked by PTX (Figure 1C). Preincubation of BMDMs with PTX prior to IVA stimulation partly blocked the inhibitory effects of IVA on OC generation, recovering multinucleated cell number (Figure 4B). Quantitative analysis showed that PTX significantly increased TRAP‐positive multinuclear cell number compared to the vehicle control in IVA‐treated groups (Figure 4C). The results suggest that IVA may inhibit OC generation in a PTX‐sensitive manner.

Diverse metabolites absorbed in the gut are associated with host energy metabolism.^18^, ^39^ AMPK plays an essential role in energy homeostasis.^40^, ^41^ AMPK also has crucial roles in regulating osteoclastogenesis. Previous reports demonstrate that activated AMPK suppresses osteoclastogenesis.^42^, ^43^, ^44^ Here, we found that IVA elicits AMPK phosphorylation (Figure 4D). The role of AMPK in the IVA‐elicited inhibitory effects on OC generation was also investigated using an AMPK inhibitor, compound C. Addition of compound C markedly reversed the inhibitory effects of IVA on OC generation (Figure 4E,F).

### 3.5 IVA attenuates OVX‐induced osteoporosis

**FIGURE 5:**
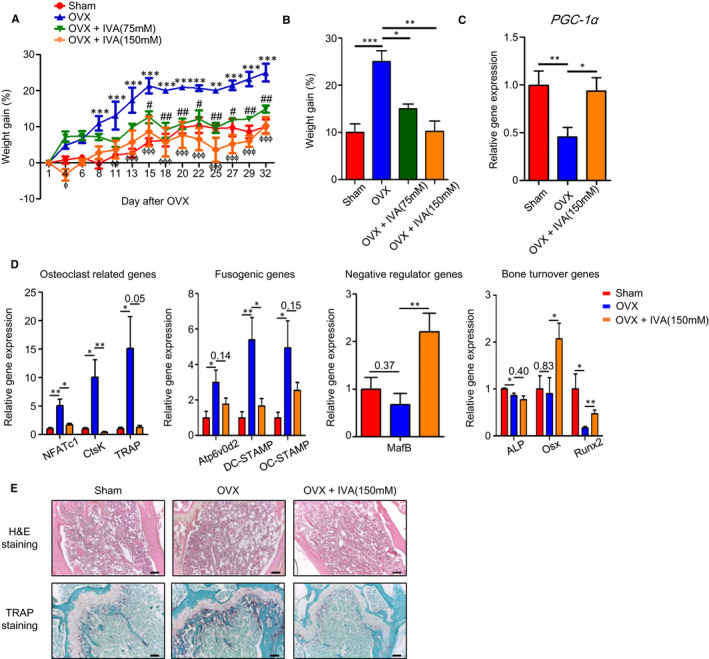
IVA shows therapeutic effects against OVX‐induced osteoporosis. A, B, Weight gain percentage was measured from the sham, OVX, OVX + IVA (75 mM), OVX + IVA (150 mM) groups for 32 days (A) and at the end of the experiment (B). C, D, Mice were sacrificed 5 weeks after OVX surgery. Femurs and tibias were harvested from each group [sham, OVX, OVX + IVA (150 mM)] to prepare RNA. qPCR analyses were performed using specific primers for *PGC‐1α* (C), OC‐related genes (*TRAF6*, *NFATc1*, and *CtsK*), fusogenic genes (*OC‐STAMP*, *DC‐STAMP* and *Atp6v0d2*), osteoclastogenesis negative regulator genes (*MafB*), bone turnover markers (*ALP*, *Osx* and *Runx2*) and *GAPDH* (D). H&E and TRAP histological staining of mouse femurs of sham, OVX, and OVX + IVA mice 5 weeks after OVX surgery (E). Data are presented as the mean ± SEM (n = 4 for A‐D). (A) Two‐way ANOVA: *P < .05, **P < .01, ***P < .001 for sham compared with OVX model, ^#^ *P* < .05, ^##^ *P* < .01 for OVX compared with OVX + IVA (75 mM), ^ɸ^ *P* < .05, ^ɸɸ^ *P* < .01, ^ɸɸɸ^ *P* < .001 for OVX compared with OVX + IVA (150 mM). (B) One‐way ANOVA: ^*^P < .05, ^**^ *P* < .01, ^***^ *P* < .001. (C, D) ^*^ *P* < .05, ^**^ *P* < .01, ^***^ *P* < .001. (E top, E bottom) Data are representative of at least three independent experiments. Scale bar; 100 μm

Since IVA inhibits OC generation induced by RANKL in vitro (Figure 2), the effects of IVA in an experimental osteoporosis model using OVX mice were examined. OVX surgery mimics postmenopausal osteoporosis showing body weight gain as well as bone density decrease and increased OC generation in bone tissue.^45^, ^46^ We also found that OVX mice gained body weight compared to control mice. Addition of IVA at the final concentrations of 75 mM and 150 mM to drinking water significantly decreased the body weight gain in OVX mice (Figure 5A). At day 32, OVX mice gain about 25% body weight; however, sham mice gain about 10% body weight. OVX mice exposed to 75 mM and 150 mM IVA gain about 15% and 10.25% body weight, respectively (Figure 5B).

Five weeks after OVX surgery, femurs and tibias were isolated and the levels of OC‐related genes were measured with qPCR analysis. Body weight increase in OVX mice is accompanied with decreased *PGC‐1α* expression, which is reversed by IVA (Figure 5C). In addition, OVX surgery significantly augmented the expression of OC‐related genes including *NFATc1*, *Ctsk* and *TRAP*, which was almost completely reversed by addition of IVA to the drinking water (Figure 5D). Fusogenic genes such as *Atp6v0d2*, *DC‐STAMP* and *OC‐STAMP* were also strongly increased by OVX surgery, and IVA exposure significantly decreased the levels of these genes (Figure 5D). However, the expression of *MafB*, a negative regulator of OC generation, was decreased by OVX and recovered by IVA exposure in OVX mice (Figure 5D). We also performed qPCR analysis of bone turnover markers such as *ALP*, *Osx* and *Runx2*. We found that addition of IVA in the OVX mice model upregulates the expression of several bone formation‐associated genes including *Osx* and *Runx2* (Figure 5D). Histological analysis with H&E staining of the bone tissue of OVX mice showed that OVX surgery markedly increased the porous area, which was strongly decreased by IVA exposure (Figure 5E top). TRAP staining demonstrated that TRAP‐positive OCs were increased upon OVX surgery; however, IVA exposure strongly decreased the numbers of TRAP‐positive OCs in the bone tissue of OVX mice (Figure 5E bottom). The results suggest that IVA shows strong inhibitory effects against OVX‐induced osteoporosis by suppressing osteoclastogenesis in vivo.

## 4 DISCUSSION

Diverse metabolites of gut microbiota can regulate many pathophysiological responses.^47^, ^48^ Among these gut microbiota‐induced metabolites, SCFAs such as acetate, propionate and butyrate have been reported to regulate various biological functions including defence activity.^8^, ^49^ A previous report demonstrated that three SCFAs, acetate, propionate and butyrate, improve systemic bone mass by attenuating OC formation in an experimental postmenopausal model as well as in a steady state.^46^ Here, we found that a BCFA, IVA, suppresses RANKL‐induced OC generation without affecting cellular viability (Figure 2). The IVA‐induced inhibitory effects on OC generation were also strongly supported by the suppressive activity of IVA on the expression of several OC‐related genes (*RANK*, *Blimp1*, *TRAF6*, *c‐fos*, *NFATc1*, *OSCAR*, *TRAP* and *Ctsk*) and fusogenic genes (*OC‐STAMP*, *DC‐STAMP* and *Atp6v0d2*) (Figure 3). Since the addition of IVA to BMDMs or pre‐OCs resulted in suppression of RANKL‐induced OC formation (Figure 2), and IVA stimulates BMDMs, causing crucial kinase activation (Akt and ERK) and chemotactic migration of the cells (Figure 1), IVA may regulate macrophage activity to suppress their differentiation into OCs.

**FIGURE 6:**
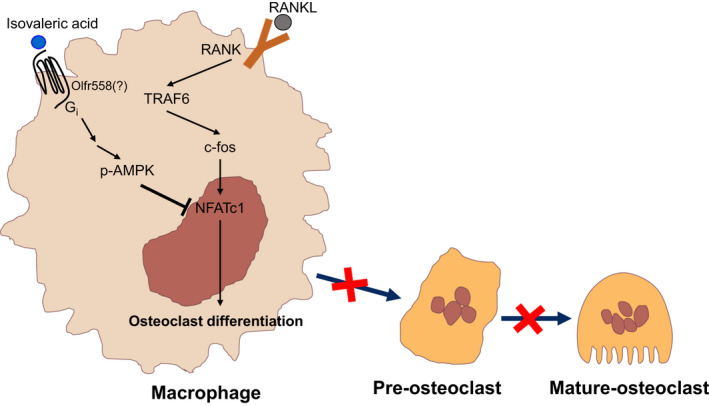
IVA blocks RANKL‐induced osteoclastogenesis by inhibiting NFATc1 through AMPK signaling

Regarding the mechanism of action of IVA on macrophage activity and differentiation into OCs, we observed that PTX significantly inhibits BMDM chemotaxis and OC formation induced by IVA (Figures 1C and 4B,C). The results suggest that IVA may act on PTX‐sensitive G_i_‐protein‐coupled receptors in BMDMs (Figure 6). In a separate experiment, we found that BMDMs express Olfr558 (data not shown), which has been reported to mediate IVA‐induced cellular responses in enterochromaffin cells.^20^ Although the functional role of Olfr558 in IVA‐induced suppression of OC formation is yet to be clarified, IVA may act on G_i_‐coupled GPCRs in BMDMs to block osteoclastogenesis (Figure 6). In a previous report, serum amyloid A inhibits osteoclastogenesis by shedding c‐fms.^23^ Unlike serum amyloid A, IVA did not induce shedding of c‐fms (data not shown), suggesting a different mode of action to suppress OC formation. Cellular signalling pathways involved in the IVA‐induced suppression of OC formation were analysed. IVA‐inhibited osteoclastogenesis was not affected by H89 (Figure 4A), ruling out a possible role of the cAMP/PKA pathway in the process. However, IVA markedly caused AMPK phosphorylation, and an AMPK inhibitor, compound C, significantly reversed the inhibitory effects of IVA on OC formation (Figure 4D‐F), suggesting that the AMPK pathway is required for the inhibition of osteoclastogenesis by IVA (Figure 6). On the regulation of AMPK activity by GPCRs, many different GPCRs were previously reported to regulate AMPK activity. Several G_s_‐coupled receptors including the β‐adrenoreceptor, several G_i_‐coupled receptors including the formyl peptide receptor and G_q_‐coupled receptors can induce AMPK activation.^50^ On the regulatory mechanism of AMPK by GPCR signalling, the G_s_/adenylate cyclase/PKA/LKB1 cascade has been reported to cause phosphorylation of the Thr 172 residue of AMPK.^51^ The G_q_ (or G_i_)/PLC/IP_3_/Ca^2+^/CaMKKβ cascade causes phosphorylation of Thr 172 of AMPK.^52^, ^53^ The detailed molecular mechanisms involved in the regulation of IVA‐induced AMPK activity is a topic for future investigation.

In this study, we found that IVA not only stimulates chemotactic migration of BMDMs but also suppresses OC formation (Figures 1 and 2). Regarding the functional relationship between the migration of OCs (or osteoclast precursors) and regulation of osteoclast differentiation, a previous report demonstrated that two chemokines, CCL19 and CCL21, stimulate osteoclast migration and bone resorption, mediating bone destruction.^54^ In this study, however, we found that IVA stimulates migration of osteoclast precursor cells, but suppresses osteoclast differentiation. At this point, it is not easy to connect the functional relationship of these two different findings. However, according to previous reports that demonstrate the functional relationship of the gut‐bone axis,^6^, ^55^ it might be possible that IVA produced by the gut as a commensal bacterial metabolite could regulate bone metabolism leading to inhibition of osteoclast differentiation. Further studies are necessary to clarify the relationship between macrophage migration and regulation of OC differentiation.

Previously, IVA was shown to be produced from a leucine‐rich diet based on animal foods such as egg and cheese through fermentation by the human gut microbiome.^17^ Here, we observed that IVA suppresses in vitro osteoclastogenesis from BMDMs and elicits therapeutic effects against an in vivo osteoporosis model, OVX mice (Figures 2 and 5). Administration of IVA decreased the expression of OC‐related genes and reduced OC generation in OVX mice, reducing bone porous area (Figure 5). In OVX mice, IVA administration also strongly increased expression of *MafB*, a negative regulator of osteoclastogenesis (Figure 5D). However, IVA did not affect *MafB* expression from BMDMs, the precursors of OCs (Figure 3D). Since *MafB* expression was analysed from the total bone of OVX mice, and total bone contains many different cell types including osteoblasts, and diverse leukocytes, as well as OCs, the contradictory effects of IVA on the expression of *MafB* in vitro and in vivo are likely caused by the complex cell types present in total bone as well as the complex regulation of *MafB* expression in vivo. In the OVX mice, IVA administration also induced *PGC‐1α* expression (Figure 5C). Since a recent study showed that *PGC‐1α* regulates osteoclastogenesis through metabolic regulation,^56^ it would be necessary to examine the effects of IVA on the regulation of metabolic activity in OC precursors. Our results suggest that IVA, a BCFA, can be used to control osteoporosis. In a previous paper, beta‐hydroxy‐beta‐methylbutyrate, another leucine metabolite, was reported to be associated with bone growth in newborn pigs.^57^ Thus, we suggest that metabolites derived from leucine‐rich diets may have potential beneficial effects against bone metabolic disorders including osteoporosis. In addition, isovaleric acidemia is an inborn error of leucine metabolism caused by a congenital deficiency of isovaleryl CoA dehydrogenase with elevated urinary IVA metabolites. Although the clinical syndrome of isovaleric acidemia is dominated by the neurologic findings, haematologic abnormalities are frequent.^58^, ^59^ Also, the unpleasant odour of IVA in urine is typical. Our study may provide some molecular mechanisms to explain the clinical phenotype of isovaleric acidemia.

In conclusion, we found that IVA, a BCFA metabolite of a leucine‐rich diet, can regulate macrophage activity and suppress osteoclastogenesis through G_i_‐coupled GPCR and AMPK‐dependent, but PKA‐independent pathways. The therapeutic effects of IVA against an experimental osteoporosis OVX model suggest that IVA and a leucine‐rich diet, which can be used to produce IVA, can be considered as a potentially new approach against OC‐related bone diseases.

## CONFLICT OF INTEREST

The authors confirm that there are no conflicts of interest.

## AUTHOR CONTRIBUTION

**Kwang Min cho:** Conceptualization (supporting); Data curation (lead); Formal analysis (lead); Investigation (lead); Writing‐original draft (lead). **Ye Seon Kim:** Data curation (supporting); Investigation (supporting). **Mingyu Lee:** Data curation (supporting); Formal analysis (supporting); Investigation (supporting). **Ha Young Lee:** Conceptualization (supporting); Data curation (supporting); Formal analysis (supporting); Writing‐review & editing (supporting). **Yoe‐Sik Bae:** Conceptualization (lead); Funding acquisition (lead); Project administration (lead); Resources (lead); Writing‐review & editing (lead).

## Data Availability

The data that support the findings of this study are available from the corresponding author upon reasonable request.
